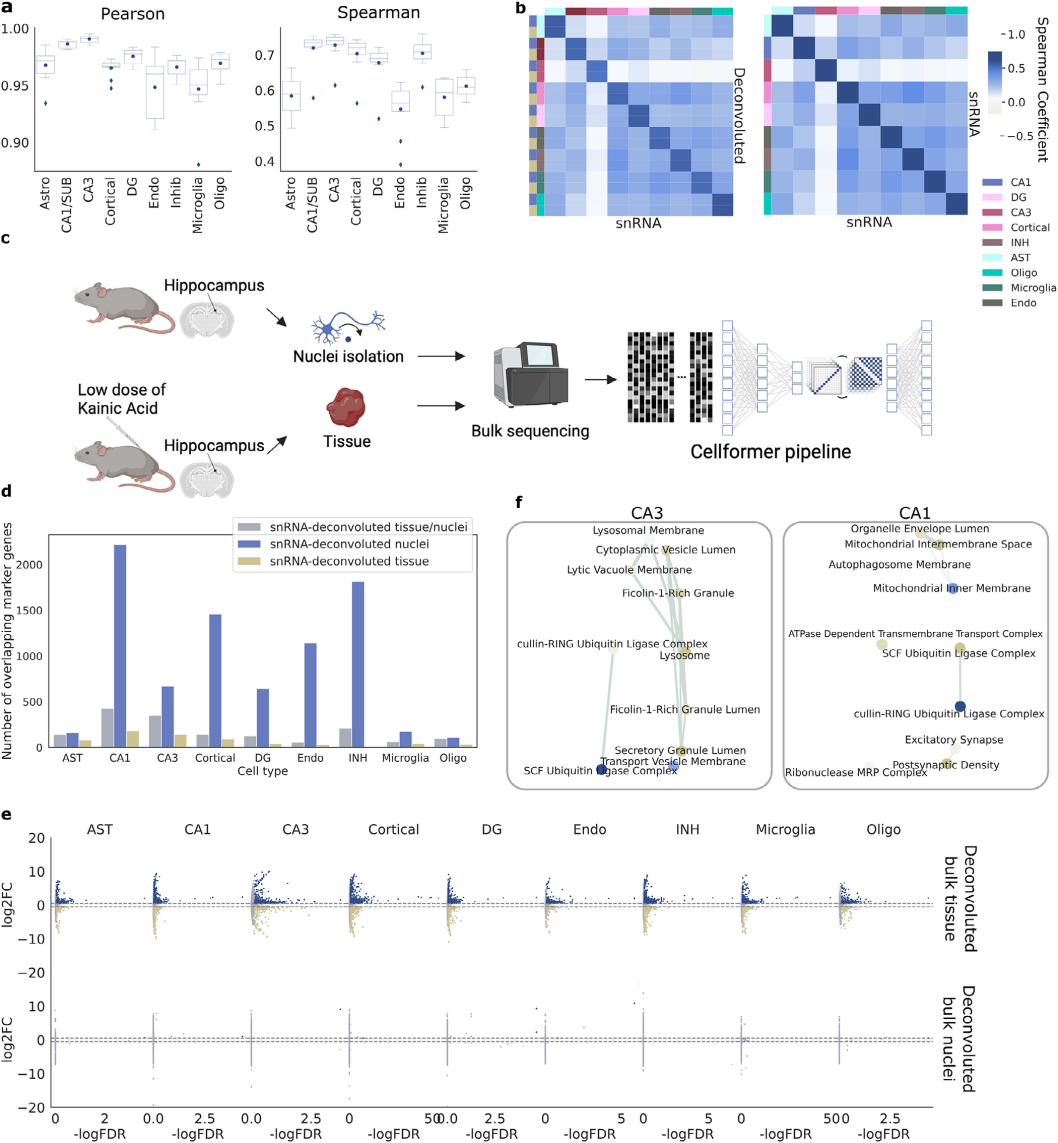# Deep learning resolves cell‐type specific transcriptomes and unveils synaptic changes associated with neurodegeneration

**DOI:** 10.1002/alz.087057

**Published:** 2025-01-03

**Authors:** Eloise Berson, Amalia Perna, Thanaphong Phongpreecha, Nima Aghaeepour, Thomas J. Montine

**Affiliations:** ^1^ Stanford, Palo Alto, CA USA; ^2^ Stanford University, Stanford, CA USA; ^3^ Department of Pathology, Stanford University School of Medicine, Stanford, CA USA

## Abstract

**Background:**

Single nucleus RNA sequencing (snRNA‐seq) has revolutionized our ability to dissect transcriptional profiles in specific cell types. While nuclear sequencing enhances analysis robustness, it captures only 20‐50% of the cellular transcriptional information, limiting our comprehensive understanding of the cellular transcriptional ensemble. Therefore, we propose a computational approach to extract the cellular signal from bulk transcriptomic data from brain tissue, allowing us to investigate cell type‐specific transcriptomic programs underlying neurodegeneration.

**Method:**

We adapted Cellformer ‐ a deep learning deconvolution model for ATAC‐seq data ‐ to RNA‐seq data. We leverage an excitotoxicity mouse model to detect cell‐type specific transcriptomic responses to injury.

**Result:**

Cellformer accurately deconvoluted mouse brain bulk RNA into 9 major cell types (mean Pearson correlation of 0.97) (**Figure 1A**). Validation with single nucleus datasets reveals a significantly higher correlation (0.85) within the same cell type compared to different cell types (0.20) (P‐value < 1e‐6) (**Figure 1B**).

We applied Cellformer to bulk RNAseq data obtained from both tissue and nuclei isolated from the hippocampus of the same mouse. We compared these cell‐type‐specific transcriptomic signatures between healthy mice and those exposed to a low dose of Kainic Acid (KA), a potent toxin for excitatory neurons (**Figure 1C**). More shared information was found between snRNA and deconvoluted RNA from nuclei compared to deconvoluted tissue (**Figure 1D**). Interestingly, differential expression analysis revealed a greater effect of low‐dose KA exposure on deconvoluted bulk tissue compared to nuclei, pinpointing synaptic and lysosomal signaling to excitatory neuronal cells (**Figure 1E‐F**).

**Conclusion:**

In this study, we introduce a computational approach utilizing the Cellformer algorithm to deconvolute bulk RNA sequencing data into cell‐type‐specific profiles, enabling in‐depth analysis of bulk RNAseq datasets. We demonstrate Cellformer’s proficiency in recovering cell‐type‐specific RNA expression patterns. By comparing deconvoluted profiles between healthy and injured hippocampi, we unveil insights previously masked by the limitations of snRNA‐seq, revealing intricate synaptic signaling dynamics. Cellformer offers the unprecedented ability to investigate extranuclear signaling in neurodegeneration.